# The effectiveness and safety of acupuncture for scoliosis: A protocol for systematic review and/or meta-analysis

**DOI:** 10.1097/MD.0000000000023238

**Published:** 2020-12-11

**Authors:** Seong-Kyeong Choi, Hyo-Rim Jo, Seo-Hyun Park, Won-Suk Sung, Dong-Ho Keum, Eun-Jung Kim

**Affiliations:** a Department of Acupuncture & Moxibustion; b Department of Rehabilitation Medicine of Korean Medicine, Dongguk University Bundang Oriental Hospital, Seongnam-si, Gyeonggi-do, Republic of Korea.

**Keywords:** acupuncture, meta-analysis, randomized controlled trials, scoliosis, systematic review

## Abstract

**Background:**

Scoliosis is a disease that affects all age groups and alters the three-dimensional deviation of the spinal axis. It is diagnosed when Cobb angle presents over 10 degrees and the reasons include aging, traumatic injuries, unknown origin, and disorders of congenital, neurological, musculoskeletal, and connective tissue. Scoliosis treatments are divided into surgical and conservative options. Surgery can correct the curved spine but has associated risks and economic burden. Conservative treatments, particularly bracing, prevent the progression of scoliosis, but still remains potential ingredient of curvation and patients poor compliance. Recent studies reported that various types of acupuncture can improve the curvation and the associated pain. However, there has been no systematic review on this issue. Therefore, this study will review the effectiveness and safety of acupuncture on scoliosis.

**Methods:**

Searching randomized controlled trials about the use of acupuncture for scoliosis will be performed using multiple electronic databases, manual search, and contact to authors e-mail if needed. We will select studies by the pre-defined criteria and summarize the data on study participants, interventions, control groups, outcome measurement, adverse events, and risk of bias. The primary outcome will be the Cobb angle, which is objective, and the secondary outcomes are associated with patient-centered indices (pain, functional status, and quality of life), which are subjective and adverse events. We will use Review Manager software (Version 5.3; Copenhagen; The Nordic Cochrane Center, The Cochrane Collaboration, 2014) to perform a meta-analysis and Cochrane Collaboration “risk of bias” tools to assess the risk of bias.

**Results:**

Our study will investigate the clinical evidence on the effectiveness and safety of acupuncture on scoliosis.

**Conclusion:**

Our study will be informative to scoliosis patients, clinicians, policy makers, and researchers working with conservative management for scoliosis.

## 1 Introduction

Scoliosis is defined as a three-dimensional deviation of the spinal axis. It is diagnosed when Cobb angle of subjects presents over 10 degrees on any part of the spine (cervical, thoracic, and lumbar part).^[1]^ Scoliosis can affect all age groups.^[2]^ During the lifetime, there are various reasons that may lead to scoliosis development, including congenital disorders, neurological disorders, musculoskeletal disorders, connective tissue disorders, aging, and traumatic injuries. Furthermore, it can have an unknown origin (idiopathic scoliosis), which can make spine curved^[3,4]^ and is known to be responsible for most cases (>60%).

In age-stratified sub-groups, the prevalence of adolescent scoliosis is reportedly 4.7% to 5.2%^[5]^ with some other articles reporting a higher percentage. A study in Korea in 2009 revealed that 1532 Korean men among 28,779 had scoliosis, suggesting a prevalence of 5.3%.^[6]^ In a study in Turkey in 2020, it was reported that up to 10.4% of 1065 adolescents had thoracic scoliosis,^[7]^ and an Iranian study in 2018 revealed that 1.4% of students had definite scoliosis, and 10.4% were suspected to have mild spine abnormality.^[8]^ In adults aged 25 to 74 adults, the scoliosis prevalence was 8.3% in the United States.^[9]^

The problems caused by scoliosis are similar regardless of its etiology. Patients have leg length discrepancy, back pain, impaired seating posture, and gait imperfection, resulting in lower health-related quality of life than healthy peers. Regarding psychological aspects, they have poor self-conception, leading to a passive social life.^[10–14]^ In addition, scoliosis can give burden to their caregiver. Caregivers life satisfaction, emotion, and economical state are also affected.^[15]^ As the treatment period prolongs, the burden increases. The only way to reduce the stress is patients recovery from the condition.^[16]^

Scoliosis treatments are divided into surgical and conservative options. Surgery is needed in scoliosis occurring from congenital and neuromuscular reasons and high Cobb angle. Early onset (age less than 10 year) of spine deformity due to the congenital, neuromuscular, or idiopathic reasons can affect spinal and pulmonary growth and leads to thoracic insufficiency syndrome.^[13,17]^ Regarding high Cobb angle, adolescents with Cobb angle >45°or multiple curves^[1]^ and adults with lumbar curves >30° and/or >6 mm of lateral listhesis are the indications of surgery.^[18,19]^

Conservative therapies include observational, bracing, casting, and physiotherapy. They are usually used to prevent the progression of scoliosis. Bracing is particularly considered to be most effective and helpful for the patient^[20,21]^; therefore, 2016 guidelines of the International Society on Scoliosis Orthopedic and Rehabilitation Treatment primarily recommend bracing for adolescent idiopathic patients.^[1]^

Conventional treatments still have their limitations. Surgery can correct the curved spine, but it has associated risks^[22,23]^ and economic burden, and there is the possibility of surgical adverse effects including infection, pulmonary embolism, cardiac arrest, nerve injury, and postoperative neuropathic pain.^[24,25]^ Living with braces still remains a potential ingredient of curvation and patients poor brace compliance^[26,27]^ and can have a negative effect on patients psychological, motor, social, and school environment.^[28]^

Recent reports suggest that acupuncture can be helpful in scoliosis. Weiss et al conducted a study and described that 1 practice of acupuncture could improve surface rotation when compared to sham needle.^[29]^ Wei et al. conducted a randomized control trial (RCT) and reported that Cobb angle of scoliosis patients significantly decreased after acupotomy, Tuina, and Daoyin therapy 1 to 2 times a week for 12 months.^[30]^ Liu et al also performed acupuncture 3 times a week for 6 weeks and reported that acupuncture not only reduced the pain but also corrected the curve.^[31]^ Regarding the Vickerss suggestion about the effectiveness of acupuncture on chronic disease,^[32]^ acupuncture could be a method to treat scoliosis and prevent its consequences with high cost-effectiveness compared to other treatments.^[33]^ However, there is a paper that present opposite suggestion. Kims review regarded that acupuncture has not proven its effectiveness as actual treatment.^[20]^

For now, there is limited evidence on the effect of acupuncture on scoliosis. Therefore, this review will focus on the effectiveness and safety of acupuncture on scoliosis by measuring the Cobb angle, patient-centered outcomes, and adverse events.

## 2 Methods

### 2.1 Study design

We willconduct this systematic review (SR) in accordance with the Preferred Reporting Items for Systemic reviews and Meta-Analyses Protocols (PRISMA-P) 2015 Statement.^[34]^

### 2.2 Ethics

No ethical statement is required since there is no patient recruitment and personal information collection.

### 2.3 Study registration

The protocol was registered in Research Registry (Registration number: reviewregistry977).

### 2.4 Eligibility criteria

#### 2.4.1 Participants

Patients who were diagnosed as having scoliosis will be included. Scoliosis is a spine abnormality wherein the spine has a lateral deviation at least 10° with vertebral rotation. This SR will include all types of scoliosis regardless of the patient age and causation, including congenital disorders, neurological disorders, musculoskeletal disorders, connective tissue disorders, aging, traumatic injuries, and unknown origin.

#### 2.4.2 Types of interventions

Various acupuncture types including acupuncture, electroacupuncture, thread-embedding acupuncture, and acupotomy for improving scoliosis will be considered eligible. The use of combination therapy during acupuncture will be accepted if the only difference between groups is acupuncture. Studies that compared between acupuncture according to the treatment duration and different acupuncture points will be excluded.

#### 2.4.3 Type of studies

This review will include RCTs. If the study did not provide the description or used an incorrect randomization method, it will be excluded from the SR and meta-analysis of acupuncture for scoliosis. Case reports, observational studies, cross-sectional studies, pilot studies, and SR protocols will be excluded.

#### 2.4.4 Outcome measures

Cobb angle will be the primary outcome measure. Secondary outcome measures are to be considered depending on review findings, such as patient-centered outcomes including pain index (visual analog scale and numerical rating scale), functional status (Japanese Orthopedic Association score and curative rates), quality of life (EuroQol five-dimensional questionnaire, Short Form 36-item) score, and adverse effects.

#### 2.4.5 Language

There will be no limits on the language.

### 2.5 Information sources and search strategy

The following electronic databases will be used from their inception to February 2021:MEDLINE, EMBASE, Cochrane library, China National Knowledge Infrastructure (Chinese database), CiNii, J-STAGE (Japanese database), KoreaMed, Korean Medical Database, Korean Studies Information Service System, National Digital Science Library, Korea Institute of Science and Technology Information, and Oriental Medicine Advanced Searching Integrated System. Researchers will perform a search using terms with a combination of diagnoses (such as scoliosis, spine curvatures, idiopathic scoliosis, degenerative scoliosis, neuromuscular scoliosis, and secondary scoliosis) and treatments (the names of various acupuncture types, such as acupuncture, electroacupuncture, thread-embedding acupuncture, and acupotomy) in each databases own language. Search will be continued from relevant gray literature sources, reports, and dissertations. If needed, manual search, such as the textbooks on acupuncture and the references and contact to authors e-mail, was also done (Table 1).

**Table 1:** Search strategy for the MEDLINE via PubMed.

No.	Search terms
#1	“scoliosis”[MeSH] OR “scoliosis”[Title/abstract]
#2	“scoliosis”[MeSH] OR “scoliosis”[Title/abstract] OR “congenital scoliosis”[Title/abstract] OR “musculoskeletal scoliosis”[Title/abstract] OR “connective tissue disorder”[Title/abstract] OR “degenerative scoliosis”[Title/abstract] OR “traumatic scoliosis”[Title/abstract] OR “idiopathic scoliosis”[Title/abstract]
#3	“acupuncture”[MeSH] OR “acupuncture treatment”[MeSH] OR “acupuncture therapy”[MeSH] OR “electroacupuncture”[MeSH] OR “thread-embedding acupuncture”[MeSH] OR “acupotomy”[MeSH] OR “acupuncture”[Title/abstract] OR “acupuncture treatment”[Title/abstract] OR “acupuncture therapy”[Title/abstract] OR “electroacupuncture”[Title/abstract] OR “thread-embedding acupuncture”[Title/abstract] OR “acupotomy”[Title/abstract] OR randomized controlled trial∗[Title/abstract] OR clinical trial∗ [Title/abstract] OR random∗ [Title/abstract]
#4	#1 AND #2 AND #3

### 2.6 Study selection

**Figure 1:**
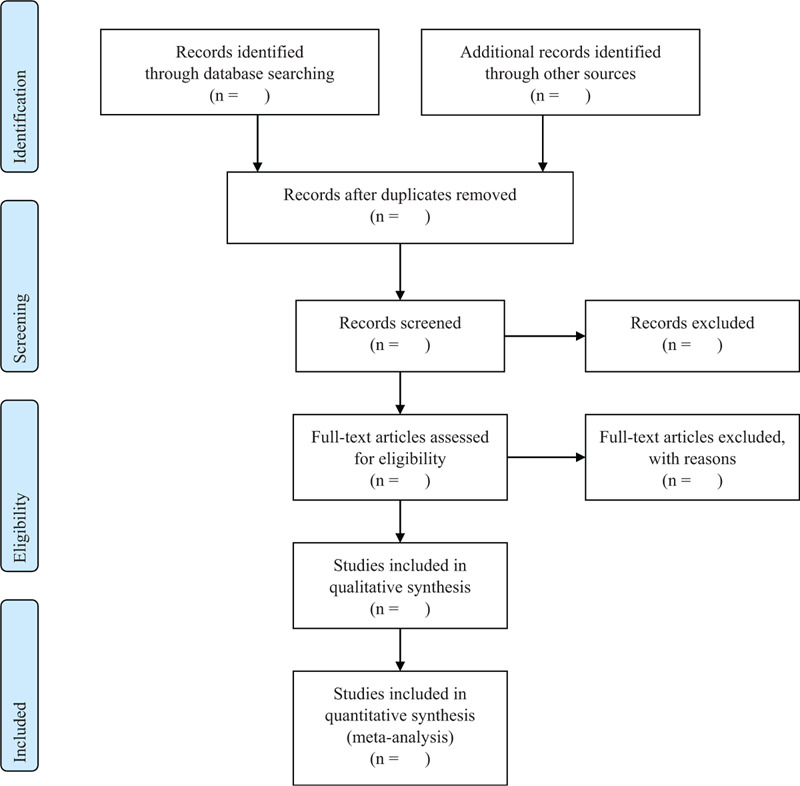
PRISMA flow diagram.

After 2 researchers are briefed about the designed qualification, each individual will independently screen the output based on the titles, abstracts, and full-text (if needed) to exclude duplicates and irrelevant reports. And then, the 2 researchers will review the studies by reading their full-texts. Disagreement between the 2 researchers will be resolved through discussion. If not possible, a third reviewer will include to mediate it (Fig. 1).

### 2.7 Data management

Researchers will use the Endnote X9 to manage the studies.

### 2.8 Data extraction

Two reviewers will gather data including patient characteristics, control characteristics, interventions, outcome measures, results, and information indicative of study quality. Any disagreement of opinion between the 2 reviewers will be solved by discussion. If a discrepancy still persists, a third reviewer will include to resolve it. If relevant data are not usable, the original author will be contacted to obtain the missing data by e-mail. If the author or data are no available, we will exclude those data and describe the reason on the paper.

### 2.9 Data synthesis and analysis

The changes from baseline in the included RCTs will be used to perform meta-analysis with Review Manager software (Version5.3; Copenhagen; The Nordic Cochrane Center, The Cochrane Collaboration, 2014). The mean difference and 95% confidence intervals (CI) in the same outcome measure, and the standardized mean difference and 95% CI in the different outcome measure will be calculated to estimate the effect. The heterogeneity assessment will be calculated by Chi-Squared and *I*-squared and be interpreted as follows; unimportant heterogeneity, 0% to 40%; moderate heterogeneity, 30% to 60%; substantial heterogeneity, 50% to 90%; and considerable heterogeneity, 75% to 100%. If possible, the subgroup analysis will be conducted based on different intervention and control interventions. If needed, the subgroup analysis with different population and the sensitivity analysis will be performed additionally. If quantitative synthesis is not possible, a narrative synthesis will be conducted based on available data.

Regarding publication bias, it is planned to use Funnel-plot when there are more than 10 identified studies in the meta-analysis. To rate the quality of evidence for each outcome, we will use the Grading of Recommendations Assessment, Development and Evaluation (GRADE) method.

### 2.10 Risk of bias assessment

Cochrane Collaboration “risk of bias” (6 domains: sequence generation, allocation concealment, blinding of participants, blinding of outcome assessors, incomplete outcome data, and selective outcome reporting) tool will be used^[35]^ to access the risk of bias by the 2 reviewers independently as high/low/unclear. Dissensus will be solved by discussion, and a third person will be brought on board to arbitrate if no consensus is reached.

## 3 Discussion

Scoliosis is a condition that affects a persons life by causing pain and morphological change. Treatments with conventional and surgical methods are helpful but have their limitations. Recently, some reports have suggested that using acupuncture for scoliosis could help with both organic and functional aspects. However, there is no SR on this issue so far. We anticipate that our study will provide the clinical evidence on the effectiveness of acupuncture on scoliosis. We hope that it will be useful a resource for health policy makers, practitioners, patients, and researchers with their further research on scoliosis.

## Author contributions

**Conceptualization:** Won-Suk Sung, Eun Jung Kim.

**Funding acquisition:** Eun Jung Kim.

**Investigation:** Seong-Kyeong Choi, Hyo-Rim Jo.

**Methodology:** Seo-Hyun Park, Won-Suk Sung.

**Project administration:** Dong-Ho Keum, Eun Jung Kim.

**Supervision:** Eun Jung Kim.

**Writing – original draft:** Seong-Kyeong Choi, Won-Suk Sung.

**Writing – review & editing:** Seo-Hyun Park, Won-Suk Sung, Eun Jung Kim.
